# Supplementary material for: Modeling double strand break susceptibility to interrogate structural variation in cancer
Source: Genome Biol. 2019 Feb 8;20:28. doi: 10.1186/s13059-019-1635-1 (PMC6368699; doi:10.1186/s13059-019-1635-1)
Supplement: Supplementary file 1 — Figure S1. Correlations between DSB profiles from three DSB HTS experiments from three different cell types at 50 kb resolution. Figure S2. Chromatin and genomic features are intercorrelated. Figure S3. Correlations between individual genomic features and DSB frequency from NHEK DSBCapture data. Figure S4. Hi-C eigenvector data improves the random forest model for DSB frequency. Figure S5. Predicted versus observed values for one third of the NHEK DSBCapture data where the random forest model was trained on the other two thirds of the data. Figure S6. ICGC SV types and cohorts. Figure S7. TCGA CNV data across cancer types. Figure S8. Correlation between predicted DSB values and observed SV breaks. Figure S9. Correspondence between SV and DSB shown for NHEK DSBCapture data and ICGC pancancer SV calls. Figure S10. TCGA CNV breakpoint enriched regions have elevated DSB frequencies in blood cancers. Figure S11. Inference of positively and negatively selected regions in ICGC breast cancers. Figure S12. Chromatin state enrichments for d-score classes. Figure S13. Finding the best-fit distribution for SV breakpoint frequencies. Figure S14. Finding the best-fit distribution for predicted DSB values. Figure S15. Finding the best-fit distribution for d-score frequencies. (DOCX 12760 kb) [file 13059_2019_1635_MOESM1_ESM.docx]

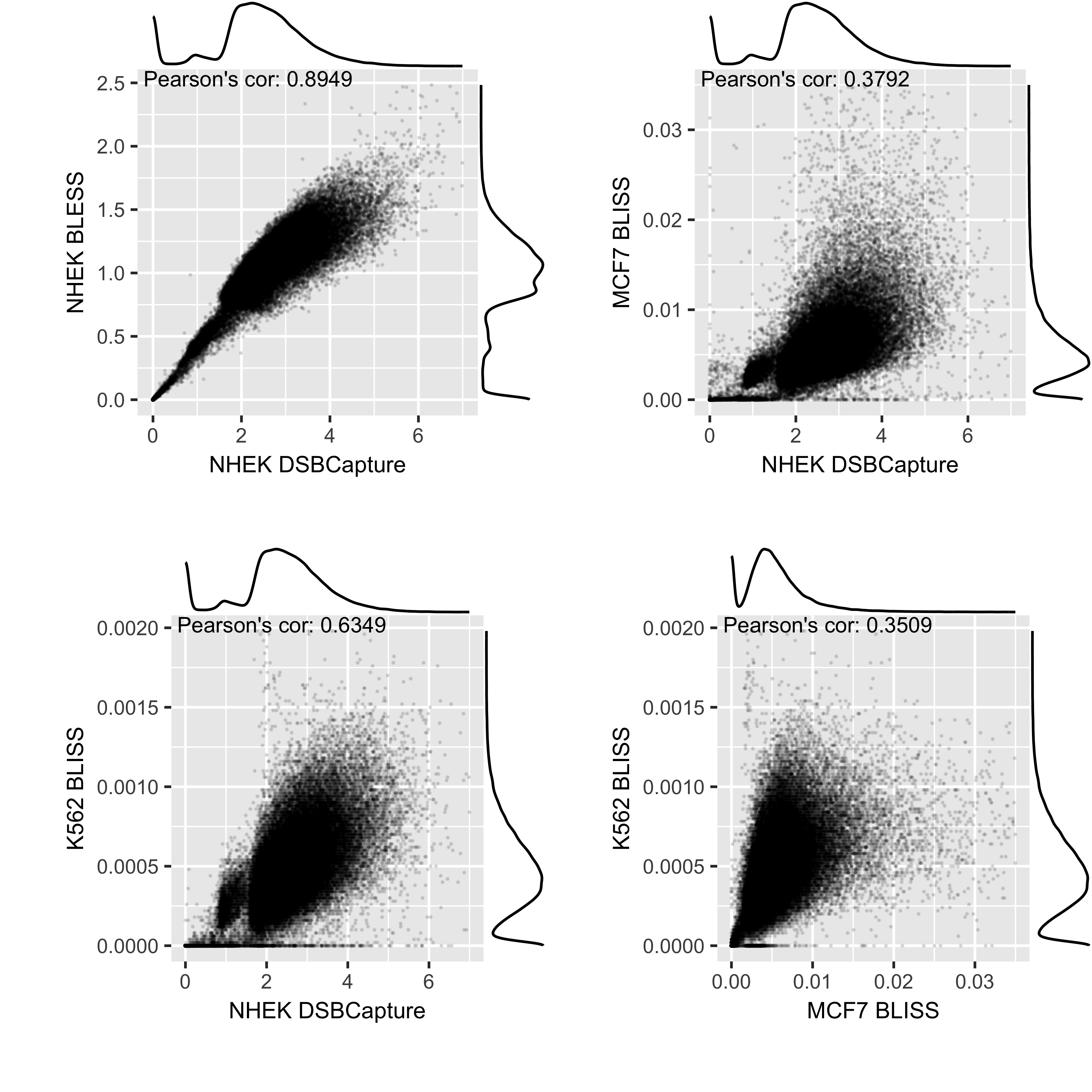


**Fig S1:** Correlations between DSB profiles from three DSB HTS experiments from three different cell types at 50kb resolution. All correlations are significant with p-values < 2.2e-16. It can be seen from the scatter plots that DSB profiles are cell-type specific and there are many differences in DSB frequency between cell types.

**Fig S2**: Chromatin and genomic features are intercorrelated. Correlation matrix between eleven features used to model DSB frequency at 50kb resolution. While G-quadruplex density and H3K9me3 are independent, with low correlation to any other feature, the other chromatin features form clusters.


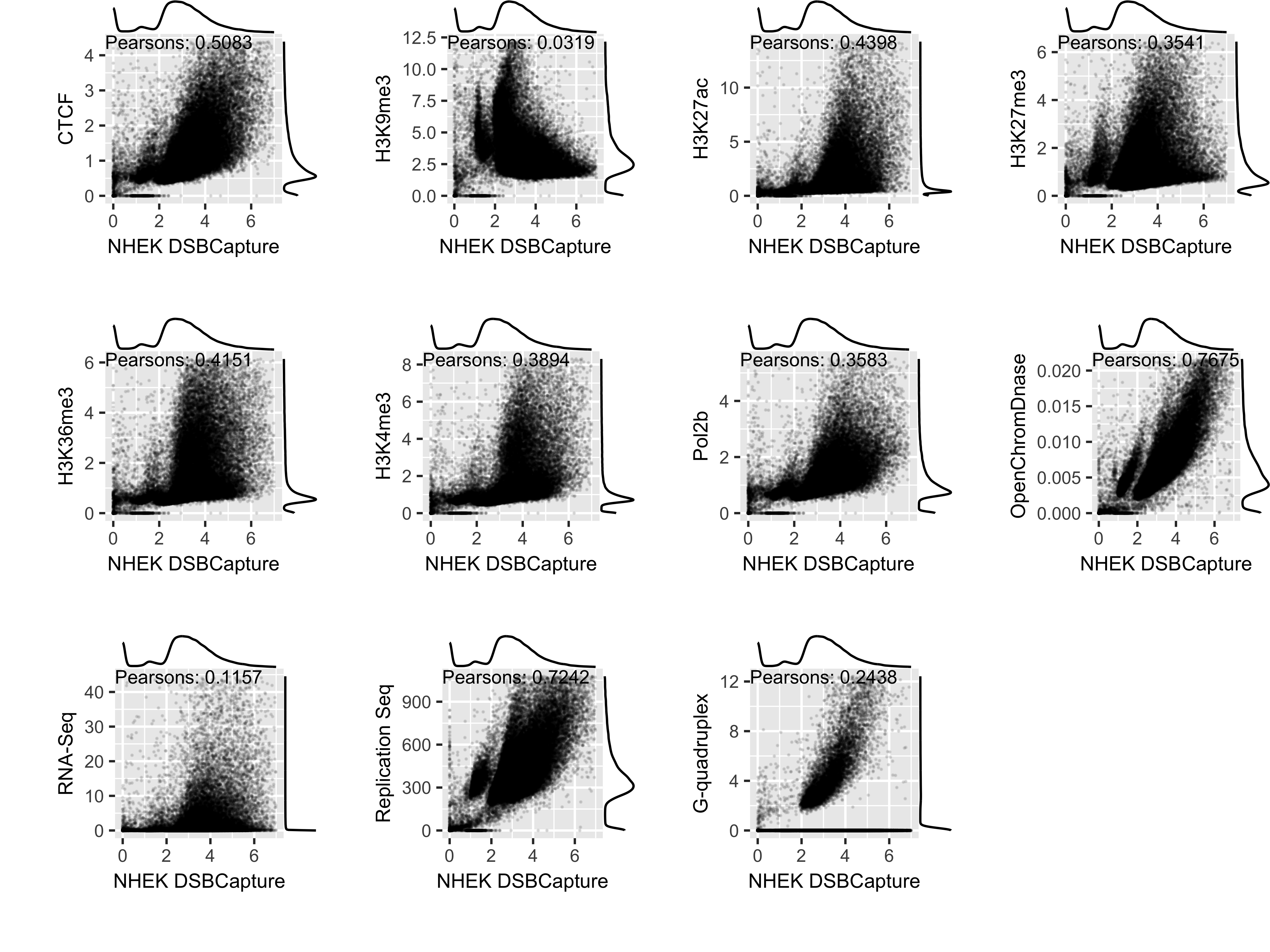


**Fig S3**: Correlations between individual genomic features and DSB frequency from NHEK DSBCapture data. It can be seen that individually, features cannot be used to predict DSB propensity as successfully as they can be used in combination through random forest models. All correlations are significant with p-values <2.2e-16, except H3K9me3, which has p-value=2.038e-15.


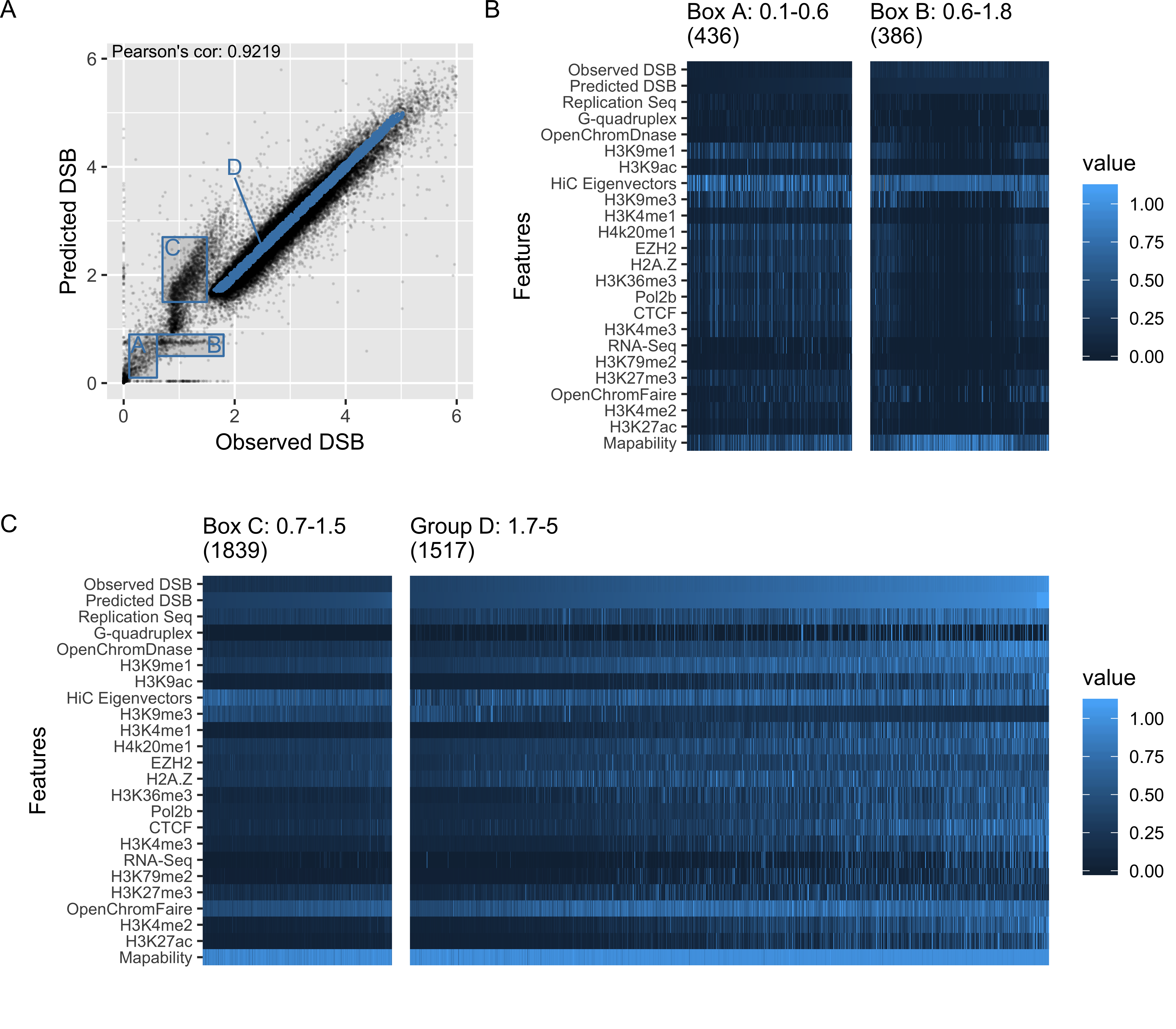


**Fig S4**: Hi-C eigenvector data improves the random forest model for DSB frequency. A) The predicted versus observed DSB frequency from the NHEK DSBCapture data for each 50kb region. The boxes A, B, and C highlight clusters of regions with poor predicted DSB frequencies, while group D contains randomly chosen points along the spectrum of DSB frequency values for the majority of the genome. B-C) Heatmaps of feature variables for each group A, B, C, and D. The numbers following group names are the observed DSB values used to define the boxed groups, and the number of regions per group is in parenthesis. Each chromatin feature was normalized as in Figure 3, with the 1^st^ to 99^th^ quantiles set to values between 0 and 1, and the 100^th^ percentile to 1.1. The columns are ordered by observed DSB frequency, shown on the top row, and the third to second to last row are ordered by variable importance. Notably, the HiC eigenvectors appear as the 6^th^ variable and distinguish regions in Box B and Box C from those in group D.


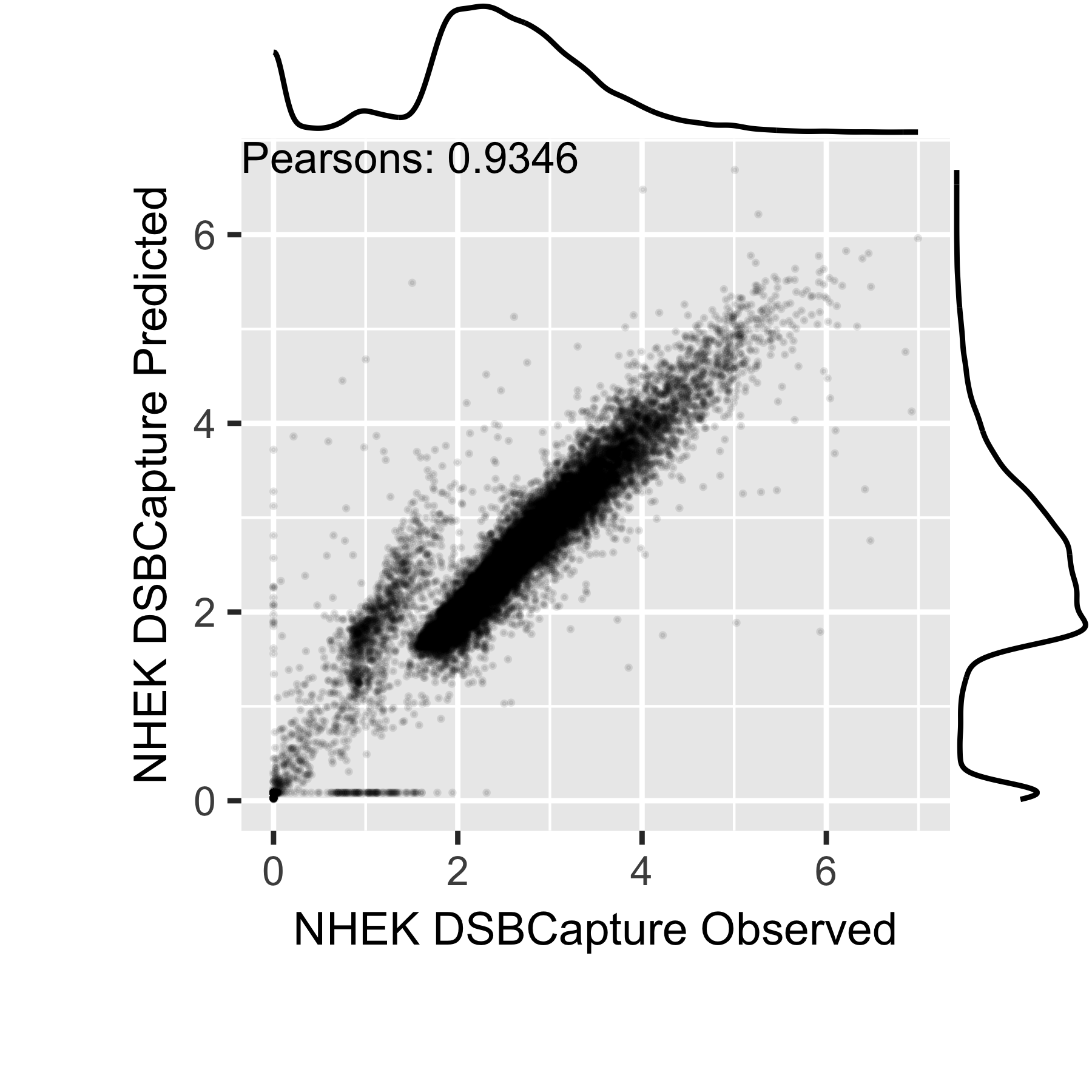


**Fig S5**: Predicted versus observed values for one third of the NHEK DSBCapture data where the random forest model was trained on the other two thirds of the data. The Pearson’s correlation is comparable to the results when the random forest is training on all 50kb bins of the genome.


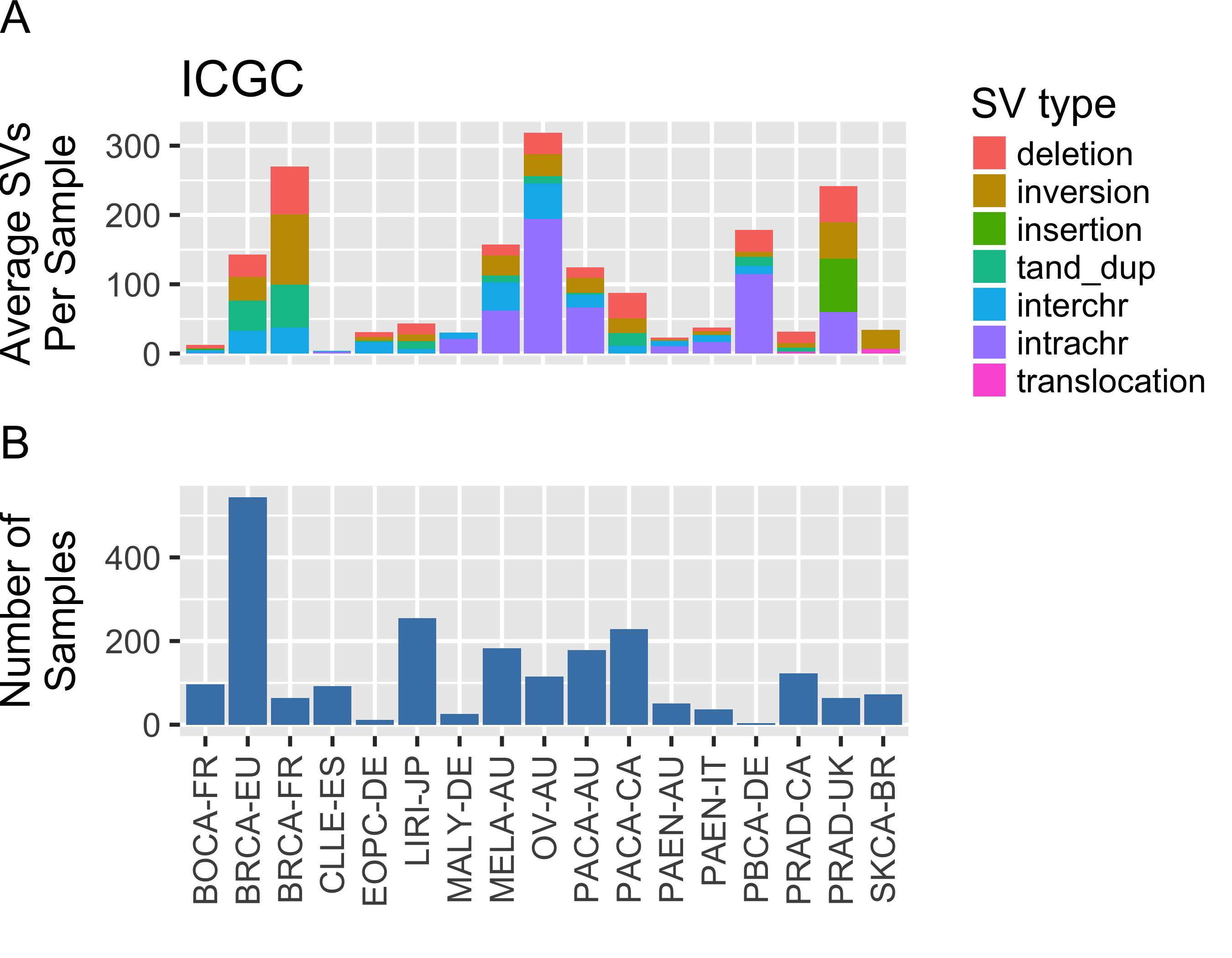


**Fig S6**: ICGC SV types and cohorts. A) The average number of SVs per patient for each cohort for seven SV types. Interchr and intrachr SVs are rearrangements either on a single chromosome or involving multiple chromosomes, respectively, and which cannot be classified into one of the simpler classes and encompass complex rearrangements such as chromothripsis or chromoplexy. Due to differences in SV detection methods between cohorts, there are large differences in the number and spectrum of SVs detected even for the same cancer types. B) The number of patients per cohort in our study, with 17 cohorts and 2,146 patients in total. The list below contains the full cancer names for each cohort.

BOCA-FR bone cancer –Ewing Sarcoma, France

BRCA-EU breast ER+ and HER2- cancer, EU/UK

BRCA-FR breast cancer, France

CLLE-ES chronic lymphocytic leukemia, Spain

EOPC-DE early onset prostate cancer, Germany

LIRI-JP liver cancer, Japan

MALY-DE malignant lymphoma, Germany

MELA-AU skin cancer, Australia

OV-AU ovarian cancer, Australia

PACA-CA pancreatic ductal adenocarcinoma, China

PAEN-AU pancreatic cancer endocrine neoplasms, Australia

PAEN-IT pancreatic endocrine neoplasms, Italy

PBCA-DE pediatric brain cancer, Germany

PRAD-CA prostate adenocarcinoma, Canada

PRAD-UK prostate adenocarcinoma, UK

SKCA-BR skin adenocarcinoma, Brazil


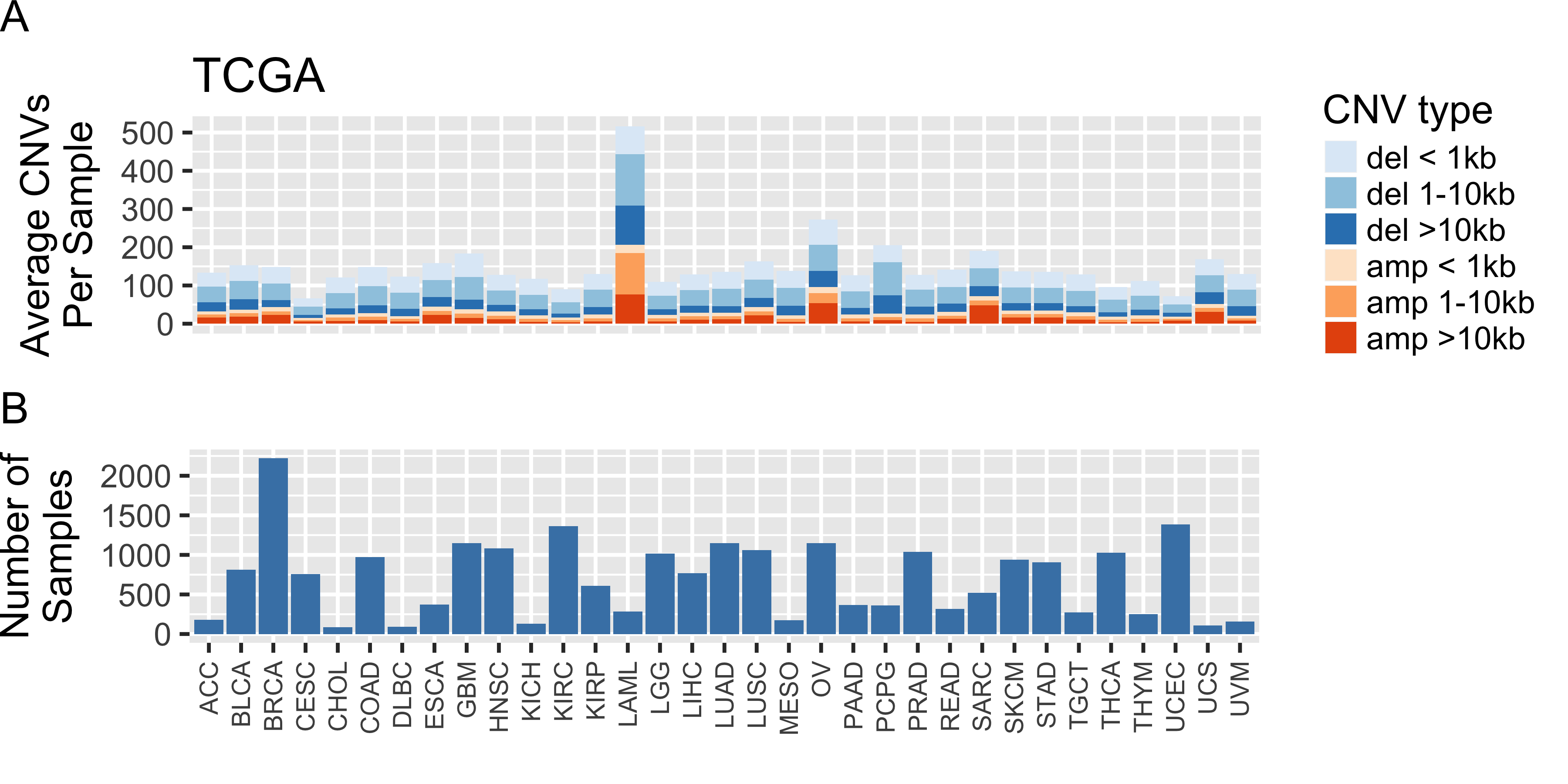


**Fig S7**: TCGA CNV data across cancer types. A) The average number of CNV per patient per cohort in TCGA, split by size. B) The number of patients per cohort in TCGA, with 23,084 patients from 33 cohorts in total. The list below shows the full cancer names for each cohort.

ACC adrnocortical carcinoma

BLCA bladder urothelial carcinoma

BRCA breast invasive carcinoma

CESC cervical squamous cell carcinoma and endocervical adenocarcinoma

CHOL cholangiocarcinoma

COAD colon adenocarcinoma

DLBC lymphoid neoplasm diffuse large B-cell lymphoma

ESCA esophageal carcinoma

GBM glioblastoma multiforme

HNSC head and neck squamous cell carcinoma

KICH kidney chromophobe

KIRC kidney renal papillary cell carcinoma

KIRP kidney renal papillary cell carcinoma

LAML acute myeloid leukemia

LGG brain lower grade glioma

LIHC liver hepatocellular carcinoma

LUAD lung adenocarcinoma

LUSC lung squamous cell carcinoma

MESO mesothelioma

OV ovarian serous cystadenocarcinoma

PAAD Pancreatic adenocarcinoma

PCPG pheochromocytoma and paraganglioma

PRAD pancreatic adenocarcinoma

READ rectum adenocarcinoma

SARC sarcoma

SKCM skin cutaneous melanoma

STAD stomach adenocarcinoma

TGCT testicular germ cell tumours

THCA thyroid carcinoma

THYM thymoma

UCEC uterine corpus endometrial carcinoma

USC uterine carcinosarcoma

UVM uveal melanoma


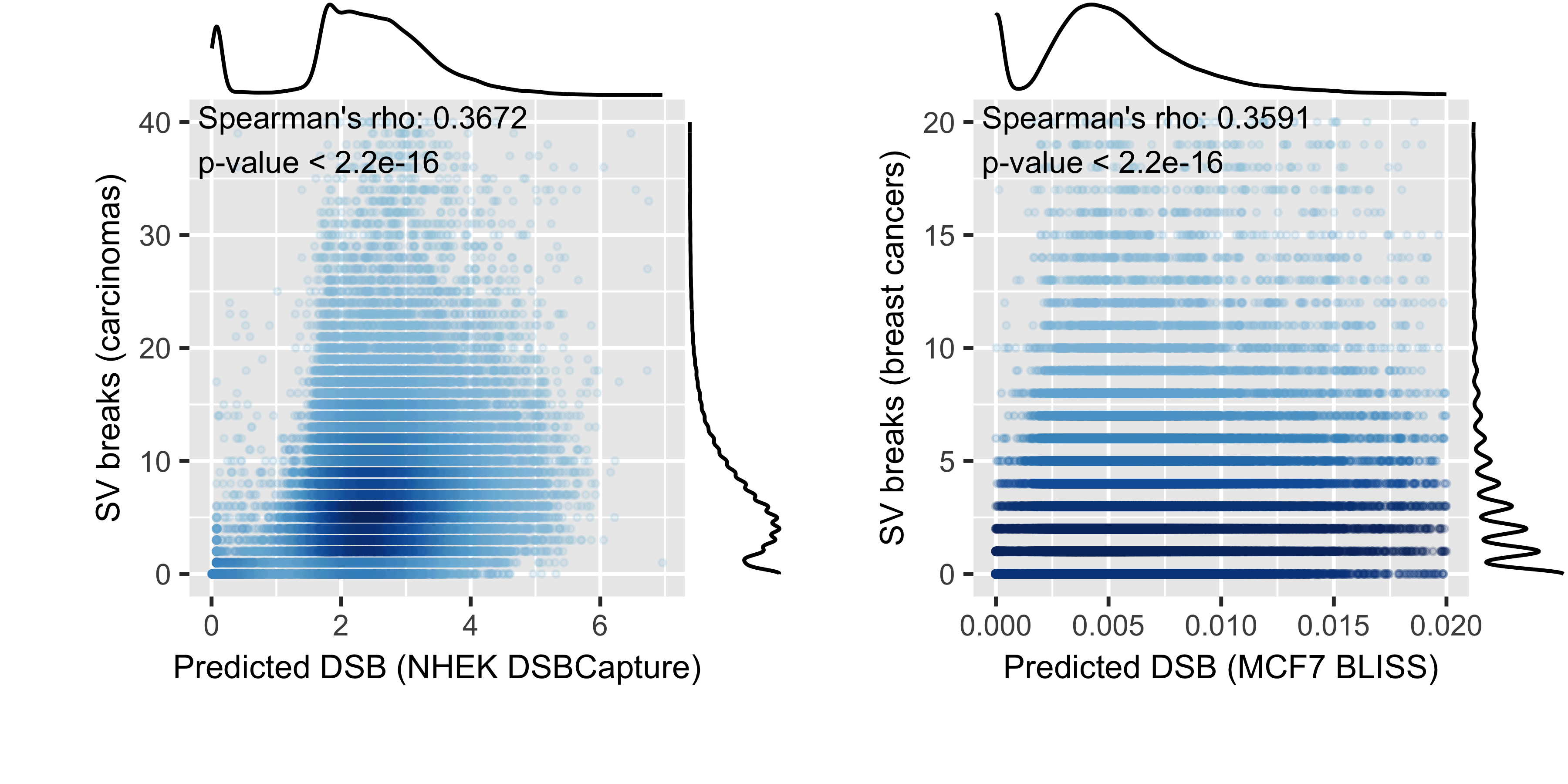
**Fig S8**: Correlation between predicted DSB values and observed SV breaks. Left) Scatter plot of ICGC carcinomas (excluding breast cancer) and predicted DSB from the NHEK DSBCapture model. Right) Scatter plot of SV breaks from ICGC breast cancers and predicted DSB from MCF7 BLISS model.


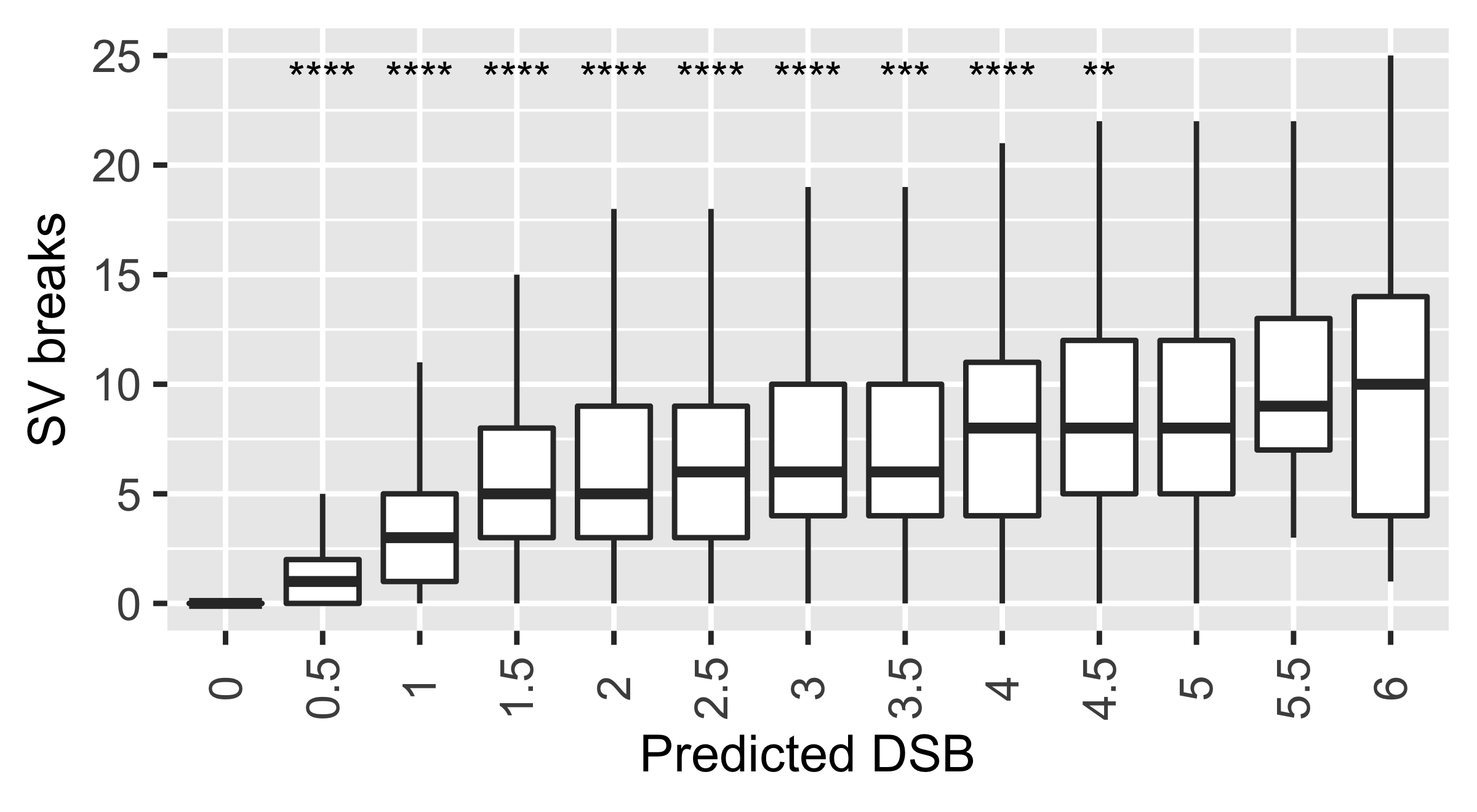


**Fig S9**: Correspondence between SV and DSB shown for NHEK DSBCapture data and ICGC pancancer SV calls. The stars indicate a significant difference in the SV breakpoint frequency between the predicted DSB frequency and the neighboring lower value (* for p<= 0.05, ** for p<= 0.01, *** for p<=1e-3, and **** for p<=1e-4). Predicted DSB values are binned by the value on the x-axis to the next value up. For example, the 0.5 box contains regions with DSB frequencies >= 0.5 and < 1.


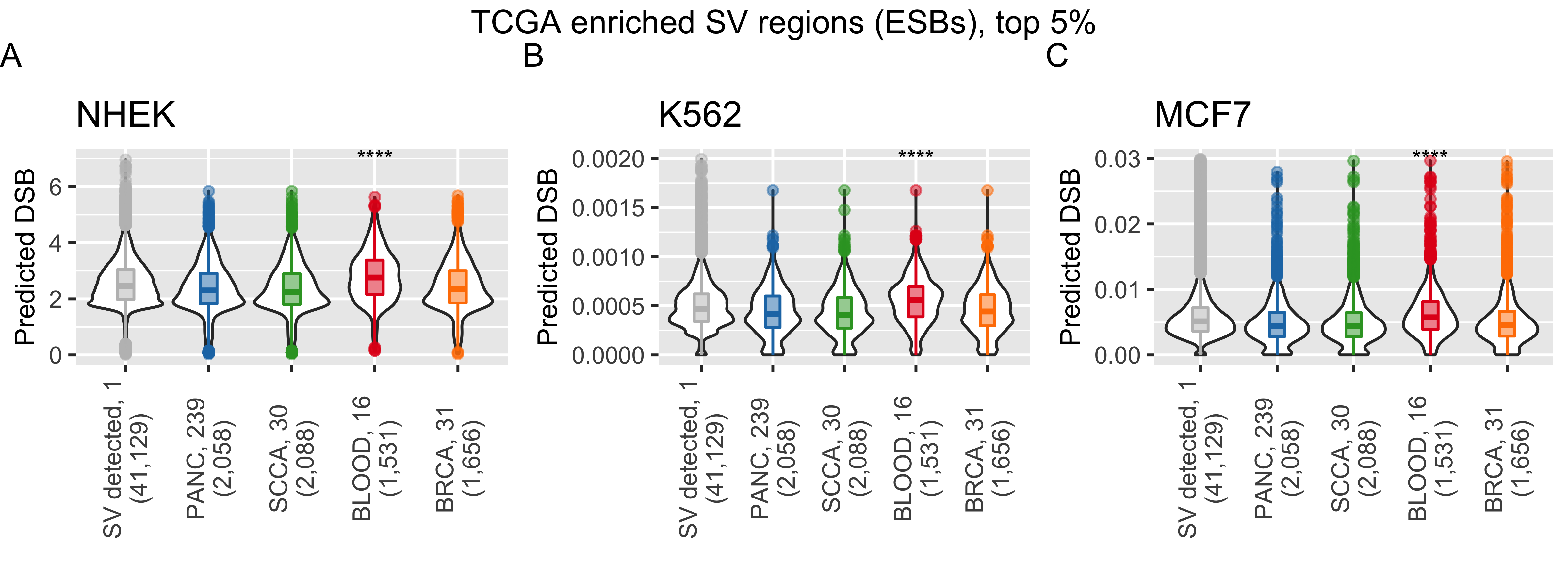


**Fig S10**: TCGA CNV breakpoint enriched regions have elevated DSB frequencies in blood cancers. Regions with SV breakpoint frequencies in the top 5% for each grouping of TCGA cancers are shown with their predicted DSB frequencies for A) NHEK DSBCapture data, B) K562 BLISS data, and C) MCF7 BLISS data. Increased predicted DSB frequency for the ESBs was tested against non-ESBs using a Wilcox ranked sum test, and the level of significance is indicated by stars (* for p<= 0.05, ** for p<= 0.01, *** for p<=1e-3, and **** for p<=1e-4).


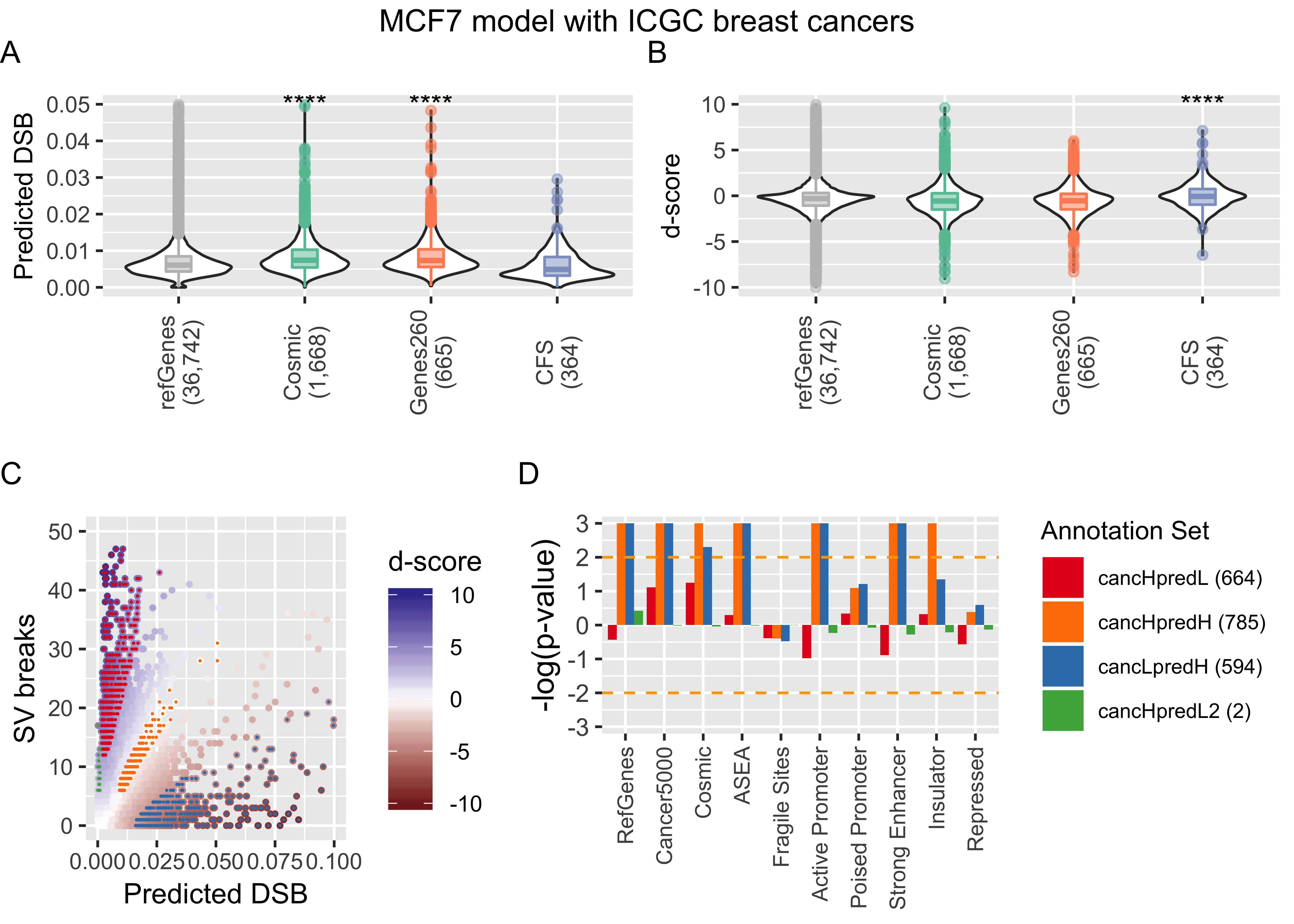


**Fig S11**: Inference of positively and negatively selected regions in ICGC breast cancers. A) The predicted DSB frequencies for regions overlapping RefSeq genes, two sets of cancer consensus genes, and common fragile sites (CFS) are shown as violin plots. The stars represent significantly higher values in the region subsets, compared to RefSeq regions, using a Wilcox ranked sum text. B) The same regions as in A), but with d-score values, a measure of the deviation of the observed breakpoint frequencies from the predicted or expected DSB frequencies. The stars represent significantly higher values in the region subsets, compared to RefSeq gene regions, using a Wilcox ranked sum text (* for p<= 0.05, ** for p<= 0.01, *** for p<=1e-3, and **** for p<=1e-4). C) Observed SV breakpoint frequencies for ICGC carcinomas (excluding breast cancer) with predicted DSB frequencies from the NHEK DSBCapture model. Each point represents a 50kb region and is coloured by its d-score. Regions were split into high (cancHpredL) and low (cancLpredH) d-score categories (d-score p-value < 0.01), a cancHpredH category, representing regions with d-scores near zero, and a cancHpredL2 category, representing low mappability regions (see methods). D) Each category was tested for enrichment of various annotations using circular permutation (see methods). The yellow dotted line marks p<0.01 significance, and the numbers in parenthesis indicate the number of 50kb regions in each category, out of 61,903 in total.


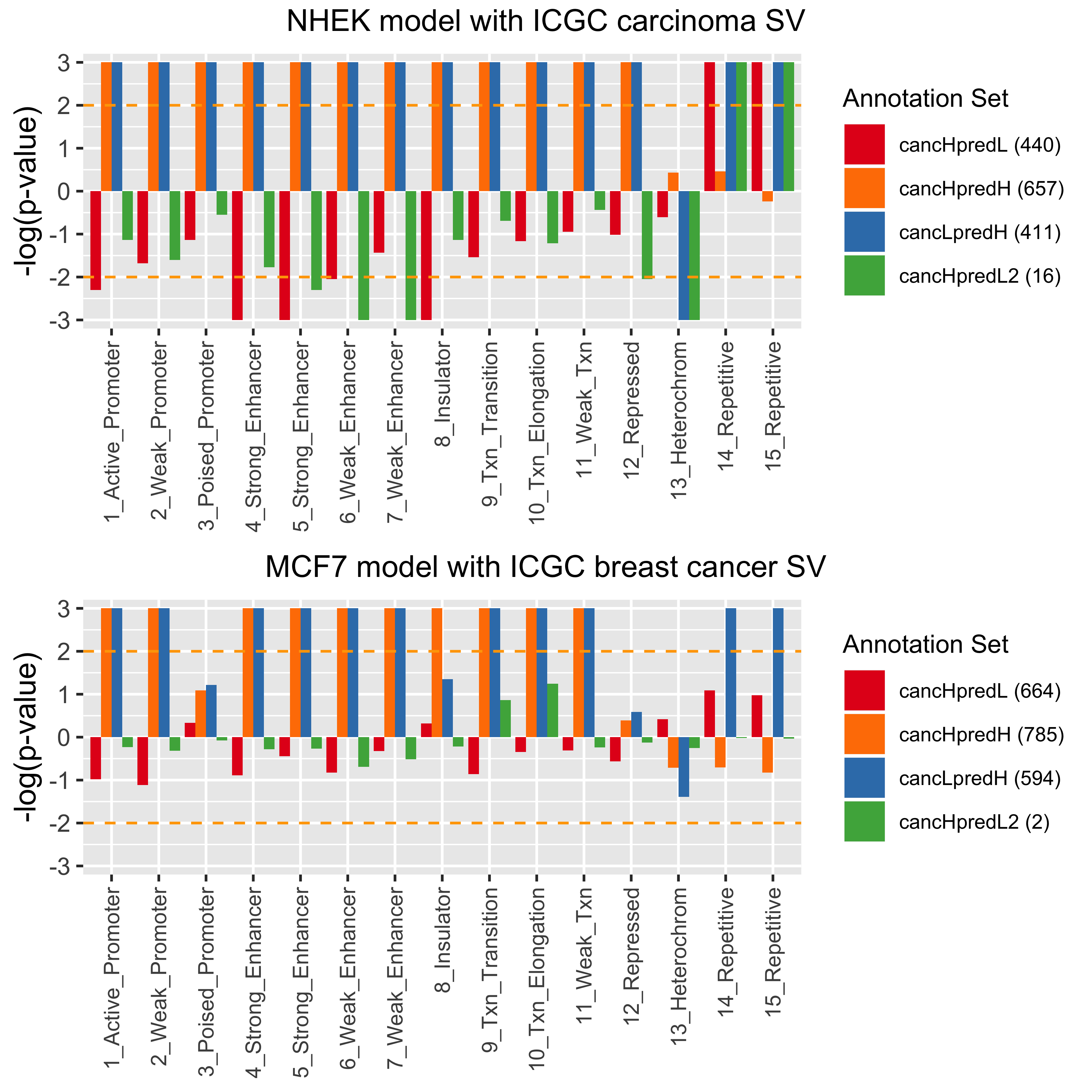


**Fig S12**: Chromatin state enrichments for d-score classes. The top panel shows the enrichment of four region classes, defined using the d-score and shown in the legend, for all 15 ChromHMM chromatin states using the NHEK random forest model and ICGC carcinoma SV breakpoint counts. The bottom panel shows the same analysis for the MCF7 model and ICGC breast cancer SV breakpoint counts. The dashed lines mark p=0.01 significance.


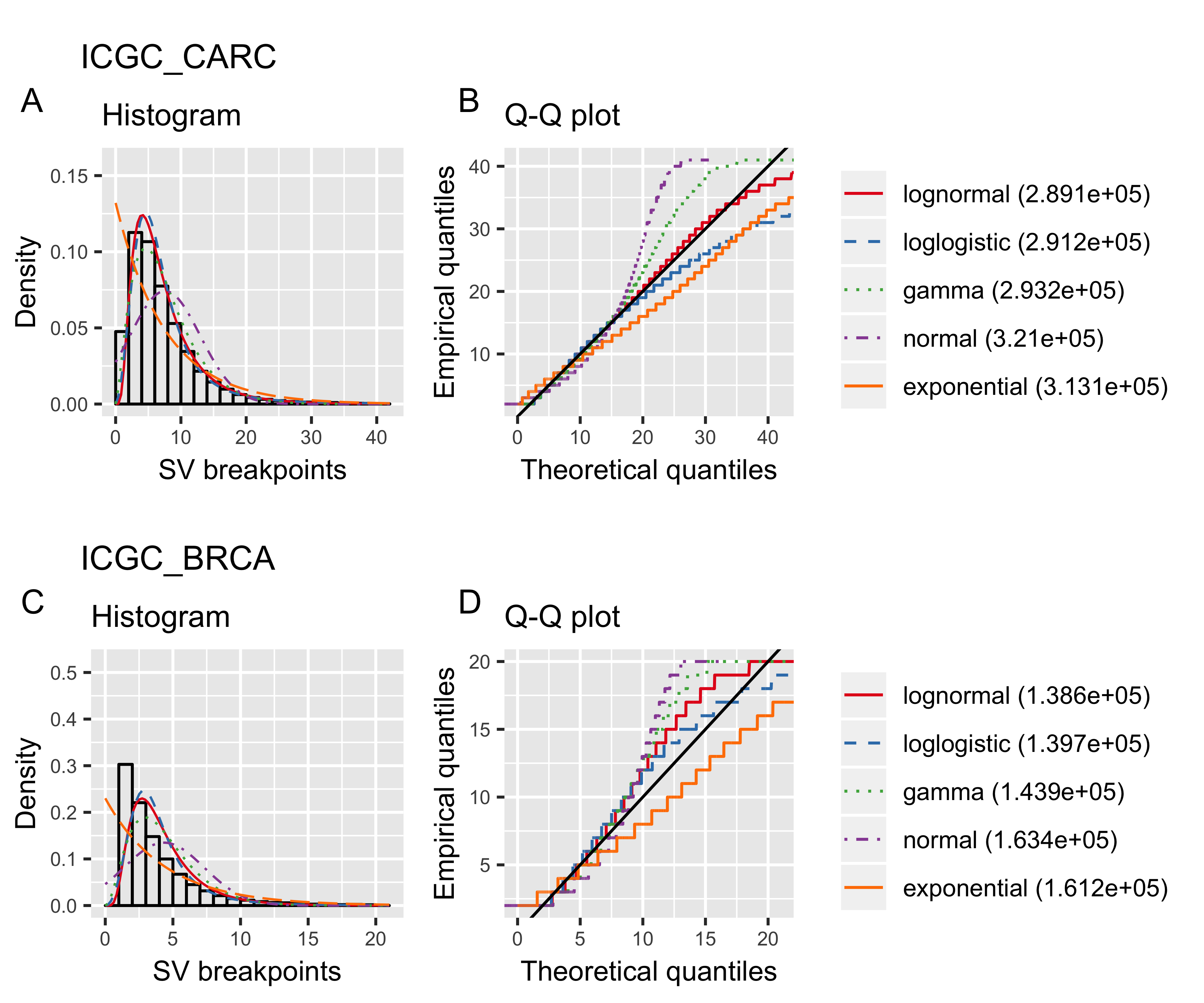


**Fig S13**: Finding the best-fit distribution for SV breakpoint frequencies. Histogram and Q-Q plots of SV breakpoint counts per 50kb region overlaid with theoretical distributions. Plots are shown for the ICGC cancer datasets: A-B) ICGC carcinomas (ICGC_CARC), and C-D) ICGC breast cancers (ICGC_BRCA). Numbers in parenthesis are the BIC score, measuring the difference between the observed and theoretical distributions. The theoretical distribution with the lowest BIC score, the lognormal in these cases, is used to assign p-values to a particular SV breakpoint count in each dataset.


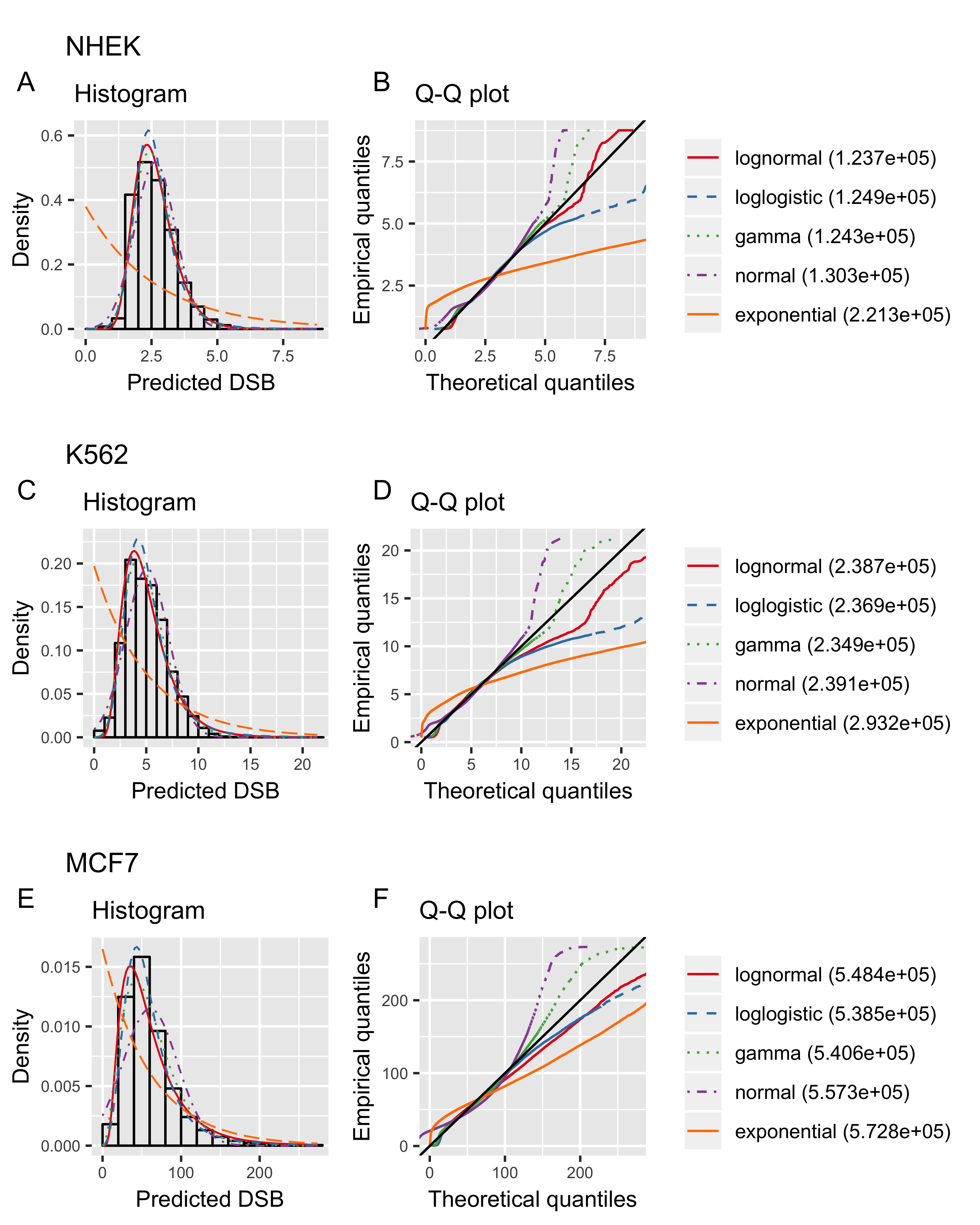


**Fig S14**: Finding the best-fit distribution for predicted DSB values. Histogram and Q-Q plots of DSB prediction values overlaid with histograms of theoretical distributions. Plots are shown for A-B) NHEK DSBCapture data, C-D) K562 BLISS data, and E-F) MCF7 BLISS data. Numbers in parenthesis are the BIC (Bayesion Information Criterion) score, measuring the difference between the observed and theoretical distributions. The theoretical distribution with the lowest BIC score for each dataset has the best fit and is used to assign a p-value to a particular DSB prediction value (the lognormal for NHEK, the gamma for K562, and the loglogistic for MCF7).


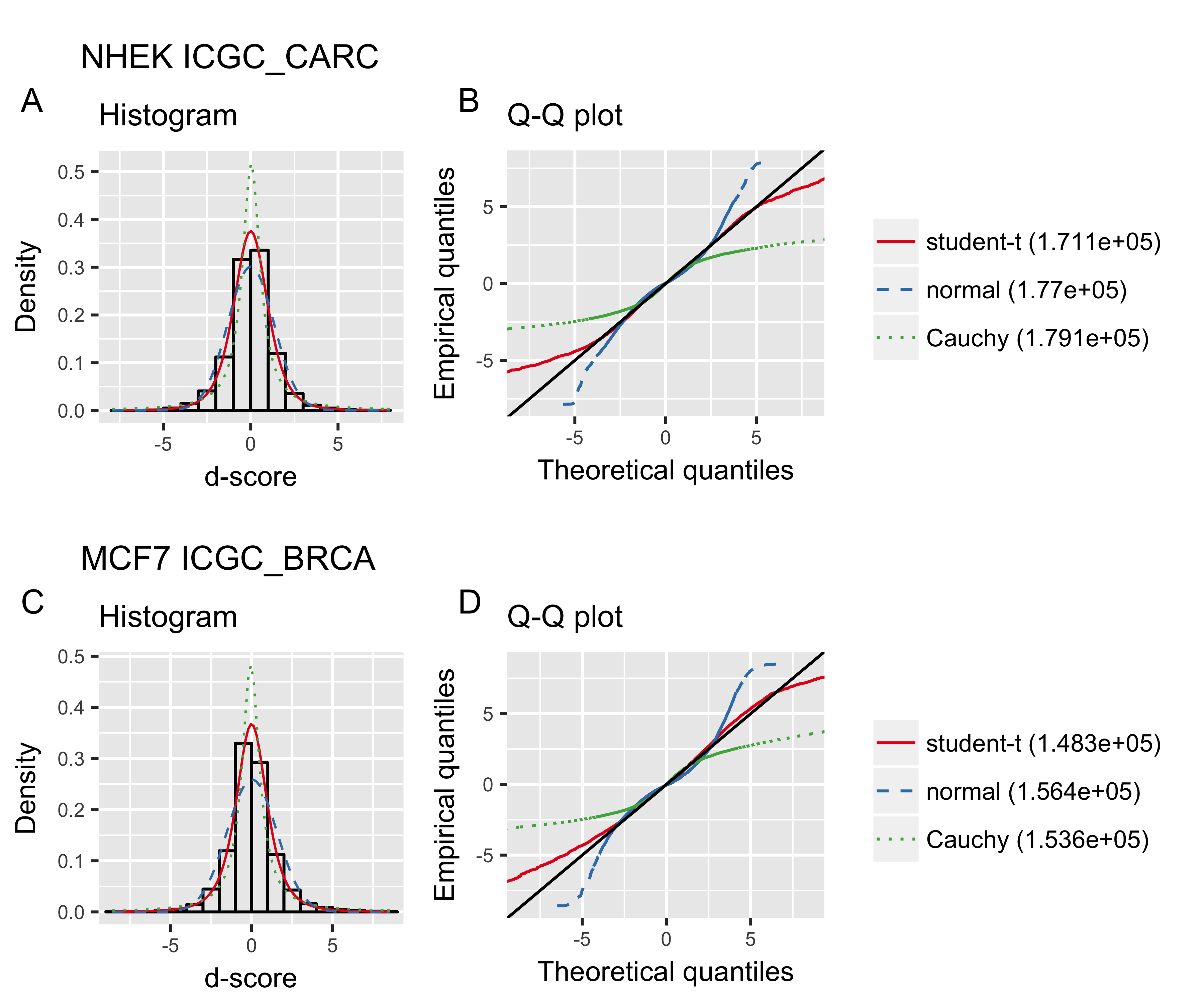


**Fig S15**: Finding the best-fit distribution for d-score frequencies. Histogram and Q-Q plots of d-scores, the log ratio of p-values for the predicted DSB and the observed SV breakpoints, overlaid with theoretical distribution densities. Plots are shown for four cancer datasets: A-B) ICGC carcinomas (ICGC_CARC) and C-D) ICGC breast cancers (ICGC_BRCA). Numbers in parenthesis are the BIC score, measuring the difference between the observed and theoretical distributions. In both cases the student-t distribution has the lowest BIC score and is used to assign a p-value to a particular d-score.
